# From Initiators to Effectors: Roadmap Through the Intestine During Encounter of *Toxoplasma gondii* With the Mucosal Immune System

**DOI:** 10.3389/fcimb.2020.614701

**Published:** 2021-01-11

**Authors:** Lindsay M. Snyder, Eric Y. Denkers

**Affiliations:** Center for Evolutionary and Theoretical Immunology and Department of Biology, University of New Mexico, Albuquerque, NM, United States

**Keywords:** *Toxoplasma gondii*, mucosal immunity, protective immunity, immunopathology, adaptive immunity, innate immunity

## Abstract

The gastrointestinal tract is a major portal of entry for many pathogens, including the protozoan parasite *Toxoplasma gondii*. Billions of people worldwide have acquired *T. gondii* at some point in their life, and for the vast majority this has led to latent infection in the central nervous system. The first line of host defense against *Toxoplasma* is located within the intestinal mucosa. Appropriate coordination of responses by the intestinal epithelium, intraepithelial lymphocytes, and lamina propria cells results in an inflammatory response that controls acute infection. Under some conditions, infection elicits bacterial dysbiosis and immune-mediated tissue damage in the intestine. Here, we discuss the complex interactions between the microbiota, the epithelium, as well as innate and adaptive immune cells in the intestinal mucosa that induce protective immunity, and that sometimes switch to inflammatory pathology as *T. gondii* encounters tissues of the gut.

## *Toxoplasma gondii* Life Cycle

*Toxoplasma gondii* is a globally distributed microorganism whose host range includes humans, domestic animals, and wildlife. The parasite is a life-threatening risk in immunocompromised individuals and a potential cause of abortion and birth defects following congenital transmission (Pfaff et al., 2007; McLeod et al., 2013). Infection is initiated in the small intestine. The parasites disseminate through the host as tachyzoites, infecting and proliferating in numerous cell types. This is followed by chronic, or latent infection, which is associated with formation of cysts containing the bradyzoite parasite form in muscle tissue and the central nervous system. *Toxoplasma* undergoes sexual reproduction in the gastrointestinal tract, but only in felines. The reason for this selectivity was a mystery until recently. Now it appears that unique aspects of lipid metabolism in cats results in unusually high systemic levels of linoleic acid that somehow signals parasite gametogenesis (Martorelli Di Genova et al., 2019). This is due to lack of intestinal delta-6-desaturase activity that is required for linoleic acid metabolism. Ingestion of oocysts (the environmentally resistant products of *Toxoplasma* sexual reproduction) shed in cat feces, as well as direct carnivorism of cysts within muscle tissue helps account for the widespread distribution of *T. gondii*.

## The Millenial Parasite

Discovered in 1908, *Toxoplasma* remained a relatively obscure parasite for most of the 20^th^ century. In large part, this was due to the asymptomatic nature of chronic infection (Dubey, 2013). With the emergence of the AIDS pandemic in the 1980s, the parasite gained widespread recognition as an opportunistic pathogen, and modern day research on *Toxoplasma* was born (McCabe and Remington, 1988). The ease of maintaining the *Toxoplasma* life cycle in the laboratory, the ability to do classical and molecular genetics on the parasite, and the rise in mouse gene knockout technology all came together to ignite an explosion in our understanding of the *Toxoplasma*–host interaction that continues to this day (Weiss and Kim, 2014).

*Toxoplasma* was one of the first microbial pathogens recognized for its ability to induce a highly polarized Th1 response that is essential for immune protection (Sher and Coffman, 1992; Denkers and Gazzinelli, 1998; Dupont et al., 2012). The parasite also played a prominent role in revealing the significance of IL-12 in triggering Th1 immunity, and conversely the key role of IL-10 in preventing these proinflammatory responses from becoming pathological (Gazzinelli et al., 1994; Gazzinelli et al., 1996). *T. gondii* was the first eukaryotic pathogen for which the importance of TLR-MyD88 signaling in immune initiation was recognized (Scanga et al., 2002; Gazzinelli and Denkers, 2006). The parasite is also a prime and possibly sole example of how an intracellular protozoan pathogen manipulates immunity through injection of host-directed effector proteins contained within parasite secretory organelles (Denkers et al., 2012; Hunter and Sibley, 2012).

The focus of this review is to assess our current state of knowledge with regard to interactions of *Toxoplasma* and the host intestinal mucosa. As the site of entry, this tissue is where the parasite establishes a foothold within the host and where it first encounters the immune system. The initial interactions occurring here are likely to determine the course of infection as the parasite spreads through the body and eventually establishes latency in the central nervous system.

## Overview of *Toxoplasma* in The Intestine

### Establishing a Foothold: Entry, Dissemination

The earliest events in establishment of *Toxoplasma* infection in the intestine are among the most crucial in determining the outcome of this host–parasite interaction, yet they are at the same time among the most difficult to study and consequently the least well understood. Use of low dose inocula that likely represent typical natural infection pushes the limits of detection, while employing artificially high infectious doses may yield results prone to artifact. Nevertheless, with current highly sensitive imaging techniques such as two-photon microscopy of living tissues, we are gaining insight into how *Toxoplasma* establishes an early foothold in its host (Luu and Coombes, 2015).

As *T. gondii* excysts from tissue cysts or oocysts in the lumen of the gut, parasites are faced with the challenge of crossing the intestinal epithelium, a barrier specialized to keep microbes out of the underlying lamina propria. The current view is that this is achieved through multiple pathways. *Toxoplasma* carries its own toolbox for cell invasion, including proteins contained within secretory granules called rhoptries and micronemes. Mechanical force for cell invasion is supplied by a parasite actin-myosin based motor. Thus, the parasite is equipped to directly enter virtually any cell type, including epithelial cells. Indeed, replicating parasites can be observed in the intestinal epithelium during early infection (Speer and Dubey, 1998). Epithelial monolayer cultures are also readily infected by tachyzoites (Briceno et al., 2016). In the intestine, rupture of these cells during the parasite lytic cycle can be expected to release tachyzoites into the underlying lamina propria. It is likely that under high infectious parasite inocula, lytic epithelial tissue destruction also enables luminal bacterial translocation triggering inflammatory gut pathology that may emerge during *Toxoplasma* infection (Heimesaat et al., 2006; Craven et al., 2012; Molloy et al., 2013).

**Figure 1:**
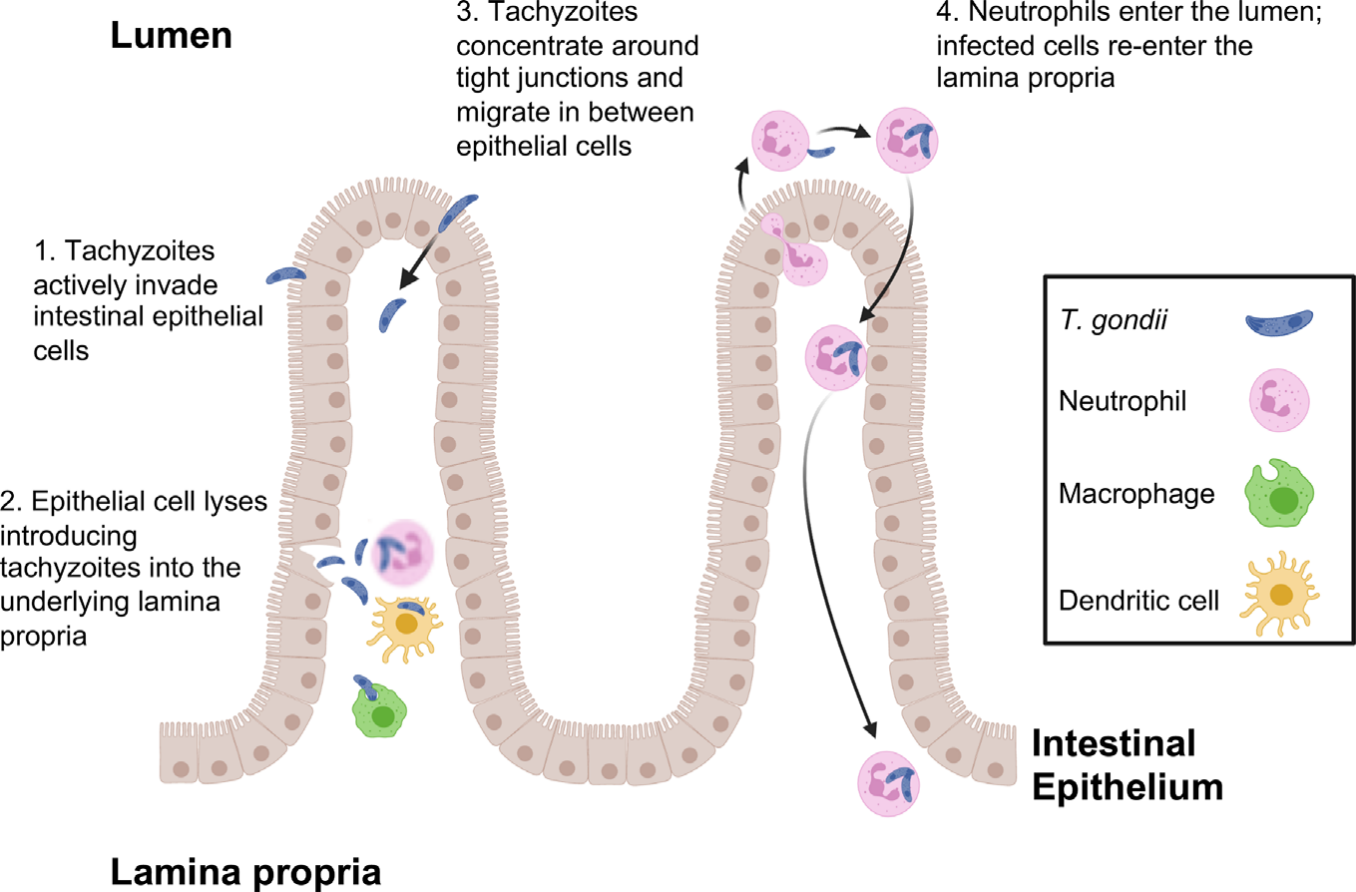
*T. gondii* pathways for crossing the intestinal epithelial barrier and early encounters with the immune system during infection. Tachyzoites cross the intestinal barrier through (1) direct invasion of intestinal epithelial cells followed by (2) cell lysis and release of parasites into the lamina propria where macrophages, dendritic cells and neutrophils are the predominant infected cell types. (3) Parasites also display the property of paracellular migration from the intestinal lumen into the lamina propria. Infection also triggers transepithelial migration of neutrophils into the intestinal lumen (4). Neutrophils in the intestine are infected, then re-enter the lamina propria at a different location. This phenomenon may account for the patchy foci of parasites that often characterize infection in the intestine.

Tachyzoites can also breach the intestinal barrier using a mechanism of transepithelial migration involving passage of the parasite between adjacent epithelial cells (**Figure 1**). Movement of *Toxoplasma* in this manner does not compromise the integrity of the epithelial barrier. Paracellular migration is linked to parasite genotype, with virulent Type I strains possessing greater ability to transmigrate than less virulent Type II and III strains (Barragan and Sibley, 2002; Barragan and Sibley, 2003). Binding between intercellular adhesion molecule (ICAM)-1 and the *Toxoplasma* microneme protein MIC2 appears to mediate this process. It has also been observed that tachyzoites co-localize with the tight junction protein occludin, which appears to facilitate paracellular transmigration of the parasite (Barragan et al., 2005; Weight and Carding, 2012). Recent data suggest that paracellular migration is facilitated by parasite secretory proteases that target tight junction proteins ZO-1, occludin, and claudin-1 (Ramirez-Flores et al., 2020).

Use of 2-photon laser scanning microscopy has more recently revealed a novel and unexpected form of *Toxoplasma* entry and spread through the intestinal mucosa. Thus, infection elicits a retrograde migration response in which large numbers of neutrophils move into the intestinal lumen. Here, or possibly in the lamina propria prior to migration, neutrophils are infected by *Toxoplasma* and they appear to subsequently establish new foci of infection throughout the intestine (Coombes et al., 2013). This may contribute to the patchiness of infection centers that are observed in the gut following oral inoculation of *Toxoplasma* (**Figure 1**).

Within a few days of infection, *T. gondii* has breached the epithelial layer and is present within the lamina propria where interactions with cells of the immune system commence in earnest. IL-12-producing mucosal dendritic cells can serve as hosts for replicating parasites (Cohen and Denkers, 2015). However, the major host cells at this location appear to be neutrophils, macrophages, and inflammatory monocytes (Gregg et al., 2013). Using the cells as Trojan horses, or possibly moving as extracellular tachyzoites, parasites begin to leave the lamina propria and disseminate throughout host tissues concurrent with the rise in adaptive immunity (Courret et al., 2006).

### Parasite Molecules That Trigger Innate Immunity: View From the Intestine

The well-known ability of *Toxoplasma* to supply a strong signal for Th1 immunity early on started a search for parasite molecules that trigger IL-12—a pursuit that continues to this day. The parasite invasion-associated protein profilin fulfills the criteria for a bona fide pathogen-associated molecular pattern (PAMP) and is recognized by Toll-like receptors (TLR) 11 and 12 (Yarovinsky et al., 2005; Kucera et al., 2010; Koblansky et al., 2012; Raetz et al., 2013a). While most studies on the influence of these TLR during *Toxoplasma* infection have been carried out in intraperitoneal inoculation models, it is also clear that profilin-TLR11 interactions are important in innate immune responses within the intestinal mucosa. Thus, there is a partial defect in generation of lamina propria Th1 cells and a partial increase in susceptibility in the absence of TLR11 (Benson et al., 2009). Parasite-induced disappearance of Paneth cells triggered by Th1 cells is also dependent upon signaling through TLR11 (Raetz et al., 2013b). Nevertheless, because humans and many other hosts of *Toxoplasma* express neither TLR11 nor TLR12, it is unlikely that profilin functions as a universal PAMP for all species the parasite infects (Gazzinelli et al., 2014). Other TLR could also be involved, although distinguishing microbiota versus parasite-driven TLR activation is a complex matter.

The dense granule protein GRA15 is a polymorphic parasite effector molecule involved in activation of NFκB from within the infected cell (Rosowski et al., 2011). In host macrophages this leads to acquisition of an M1 phenotype, including production of IL-12 (Jensen et al., 2011). Of the three major parasite strains that predominate in Europe and North America (Types I, II and III), only the Type II strain expresses active GRA15 (Rosowski et al., 2011). During oral infection, deletion of GRA15 alone does not influence mouse susceptibility or parasite replication. However, in the context of Type I ROP16, a secretory parasite kinase that activates STAT3, 5 and 6 and promotes an anti-inflammatory M2 macrophage phenotype, deletion of GRA15_II_ increases parasite number and inflammation in the intestine (Jensen et al., 2013). Thus, active GRA15 most likely exerts effects on innate immune effectors in the intestine through its ability to commandeer NFκB signaling in infected cells.

Another dense granule protein that can induce IL-12 is GRA24. This molecule is inserted into the host cytoplasm where it triggers autoactivation of mitogen activated protein kinase p38 leading to increased IL-12 gene transcription (Kim et al., 2005; Braun et al., 2013; Mercer et al., 2020; Mukhopadhyay et al., 2020). While GRA24 can drive a protective immune response in an intraperitoneal vaccination model, its role in infection of the intestinal mucosa is not yet known.

Inflammasomes have recently attracted a great deal of attention as cytoplasmic sensors of infection. This is particularly true for intracellular protozoan parasites (Zamboni and Lima-Junior, 2015; de Carvalho and Zamboni, 2020). For the case of *Toxoplasma*, inflammasome components NLRP1 and NLRP3 respond to infection resulting in IL-1*β* and IL-18 release, in turn promoting resistance to infection (Witola et al., 2011; Ewald et al., 2014; Gorfu et al., 2014). As yet unresolved are findings of others indicating that inflammasome activation only emerges as a significant factor in the absence of TLR11 signaling (Lopez-Yglesias et al., 2019). The parasite secretory molecule GRA15_II_ has been implicated in IL-1β and IL-18 production, although whether this is due to inflammasome assembly or NFκB-dependent induction of pro-IL-1β and pro-IL-18 is not clear (Gov et al., 2013). Rat macrophage pyroptosis, diagnostic of inflammasome activation, was recently found to be dependent upon GRA35, 42 and 43 (Wang Y. et al., 2019). Along parallel lines, *T. gondii*-triggered potassium efflux can act as a signal for IL-1β release, suggesting that it may drive inflammasome assembly as is known to occur in other situations (Gov et al., 2017). An increase in susceptibility was reported during oral infection of caspase1/11 knockout mice suggesting inflammasome involvement (Ewald et al., 2014). In human intestinal epithelial cells, *Toxoplasma* infection resulted in NLRP3-dependent IL-1β release that was mediated through the ATP receptor P2X7 (Quan et al., 2018). Finally, it was reported that IL-1R knockout mice display increased Paneth cell depletion associated with *T. gondii* infection, further implicating inflammasome activation (Villeret et al., 2013). The role of inflammasome activation in detecting infection in the intestinal mucosa, as well as downstream inflammation and immunity requires further attention.

Recently, the alarmin S100A11 was identified as a host molecule that triggers early immune responses in human monocytes (Safronova et al., 2019). It was also found to promote monocyte recruitment during oral infection in mice, likely through the chemokine CCL2. The S100A1 protein may function as a damage associated molecular pattern molecule released by infected cells with an important function in initiation of immunity. Rather than directly triggering IL-12 production and Th1 response initiation, this alarmin is more likely to be involved in immune recruitment during early infection. The PAMPs and DAMPs currently believed to be involved in the response to *Toxoplasma* in the gut mucosa are shown in **Table 1**.

**Table 1:** Pathogen-associated molecular pattern molecules and host danger-associated molecular pattern molecules that play a role in anti-*Toxoplasma* immunity in the gut.

PAMP/DAMP	Receptor	Downstream Function	References
Profilin	TLR11/12	Activation of DC, macrophages, and neutrophils triggering IL-12 production	(Yarovinsky and Sher, 2006; Kucera et al., 2010; Koblansky et al., 2012; Raetz et al., 2013a)
Commensal-derived molecules	TLR2	IL-12 production and enhanced type I immunity	(Benson et al., 2009)
Bacterial flagellin	TLR5	Flagellin specific CD4+ T cells that contribute to anti-*Toxoplasma* type I immunity	(Hand et al., 2012)
Commensal-derived molecules (LPS)	TLR4	Enhanced IL-12 and IFN-γ production	(Benson et al., 2009)
CpG	TLR9	Enhanced type I immunity and reduced systemic parasite burdens	(Minns et al., 2006; Benson et al., 2009)
ATP	P2X7	NLRP1, NLRP3, NLRC4 and AIM2 inflammasome activation and IFN-β production	(Quan et al., 2018)
S100A11	RAGE	Induction of CCL2 and recruitment of inflammatory monocytes	(Safronova et al., 2019)

### Intestinal Microbiota Influences Progression of Infection

Infection with *T. gondii* can result in an effective protective Th1 immune response or in fulminant and ultimately lethal inflammatory tissue destruction in the small intestine. Genetics and infectious dose play important roles in determining these polar outcomes (Liesenfeld et al., 1996; Liesenfeld et al., 1999). We now also understand that the gut microflora strongly influences each of these divergent responses.

Typically, the intestine is regarded as a location of continual, low intensity skirmishes between the immune system and normal microbiota, while overall the immune system remains tolerant to gut microbes (Sansonetti, 2011; Kayama and Takeda, 2012; Nutsch and Hsieh, 2012). *Toxoplasma* breaks this tolerance, insofar as oral infection stimulates a Th1 response to bacterial flagellin (Hand et al., 2012). Remarkably, the microbiota-specific T cells emerging during *T. gondii* infection are comparable in number to parasite-specific T cells in the intestinal mucosa. While the trigger for the breach in tolerance is not known, it may be a downstream effect of loss of Paneth cells, a rich source of antimicrobial peptides, that is driven by *Toxoplasma* infection (Raetz et al., 2013b; Burger et al., 2018).

Other studies have revealed that the intestinal microbiota exerts an important adjuvant-like effect on development of *Toxoplasma*-specific immunity. Thus, in the absence of TLR11, IL-12 and parasite-specific Th1 responses are retained—unlike the immune response following intraperitoneal parasite inoculation which is highly TLR11-dependent (Benson et al., 2009). Importantly, depletion of microflora with antibiotics abrogates this TLR11-independent response in the gut and there is a concomitant increase in susceptibility to *Toxoplasma*. The mucosal response occurring in the absence of TLR11 appears to involve the combination of TLR2, 4 and 9, receptors well known to recognize bacterial ligands. While each of these TLRs signals through the MyD88 molecule, it is known that in the absence of this signaling adaptor, Th1 responses while diminished are still retained (Sukhumavasi et al., 2008). Clearly there are other MyD88-independent pathways in the mucosal immune system that await discovery.

We also understand that perturbations in intestinal microflora play a key role in inflammatory tissue damage associated with high dose *T. gondii* infection. Similar trends in microbial dysbiosis have been observed in Crohn’s disease patients (Egan et al., 2011a; Vester-Andersen et al., 2019). After peroral infection with *Toxoplasma*, mice exhibit decreased microbial diversity, outgrowth of *γ*-Proteobacteria (including *Enterobacteriaceae*) and increased epithelial adhesion and invasion by *Escherichia coli* (Heimesaat et al., 2006; Craven et al., 2012). This culminates in extensive epithelial tissue damage and bacterial translocation into the underlying lamina propria. A direct role for gut microbes in this process is demonstrated by the fact that *Toxoplasma*-induced intestinal pathology is prevented by antibiotic administration prior to infection. There is evidence that bacterial TLR4 ligands are involved in this uncontrolled proinflammatory response (Heimesaat et al., 2007). It has also been reported that *Toxoplasma* infection elicits neutrophil migration into the intestinal lumen, generating structures that encapsulate microbiota which limits contact with damaged epithelium (Molloy et al., 2013). In addition, transfer of lamina propria CD4^+^ T cells along with intraepithelial lymphocytes from infected mice into non-infected mice drives intestinal damage that is dependent upon recipient gut flora (Egan et al., 2011b). This indicates that at least part of the inflammatory pathology is due to bacteria-specific T cells in the mucosal immune compartment. The effects of the intestinal microbiome on acute infection are well established. However, it is also possible that the microbiome influences later events in infection, for example parasite reactivation in the brain and emergence of toxoplasmic encephalitis. While corresponding effects have been characterized elsewhere (Zhu et al., 2020), this is an unexplored area in *Toxoplasma* research.

## Mucosal Immune Response During *Toxoplasma* Infection: Cast of Characters

When *Toxoplasma* enters the gut, cells of the mucosal immune system and associated tissues are rapidly alerted to infection. Some of the key cells are resident in the intestinal mucosa, others are recruited. Regardless, there is a coordinated response involving cells of innate and adaptive immunity. As outlined above, the outcome may be protective immunity and survival, or tissue pathology and death. The following summarizes the activities of cells most relevant to the course of infection in the gut.

### Epithelial Cells

The intestinal epithelium is the initial line of defense between the host and intestinal pathogens. As such, cells in this compartment are the first to encounter *Toxoplasma* in the gut. Epithelial cells include enterocytes, goblet cells, Paneth cells, M cells, and enteroendocrine cells (van der Flier and Clevers, 2009; Allaire et al., 2018). Paneth cells, a rich source of antimicrobial peptides in the intestine, decrease in number during *Toxoplasma* infection. Loss of Paneth cells has been implicated in *T. gondii-*driven intestinal dysbiosis and immunopathology. Raetz et al. showed that Paneth cells are destroyed by IFN-*γ* producing CD4^+^ T cells triggered by *T. gondii* infection (Raetz et al., 2013b). Destruction of Paneth cells is dependent upon presence of the intestinal microbiota and T cell intrinsic MyD88 signaling. Loss of Paneth cells results in impaired intestinal barrier function, *Enterobacteriaceae* outgrowth, and intestinal pathology (Raetz et al., 2013b; Burger et al., 2018). In addition to antimicrobial peptide production, Paneth cell intrinsic autophagy is important for regulating immunopathology in response to *T. gondii* infection. Loss of the autophagy protein Atg5 in Paneth cells results in severe immunopathology, loss of crypt structures, and increased host mortality, all of which are dependent upon presence of the intestinal microbiota (Burger et al., 2018). Together, these studies describe new, important roles for Paneth cell autophagy and antimicrobial peptide production in limiting immunopathology and microbiota dysbiosis driven by *T. gondii* infection.

### Intraepithelial Lymphocytes

Intraepithelial lymphocytes (IELs) are interspersed throughout the intestinal epithelium and evidence indicates they play an important role in anti-*Toxoplasma* immunity. The IEL compartment is comprised primarily of *γδ* ^+^ T cells and CD8^+^ T cells, most of which express the CD8*α* homodimer (Guy-Grand et al., 1991; Cheroutre et al., 2011). A homeostatic function in the intestine is often ascribed to this compartment. Nevertheless, the complexity of the IEL population suggests their role extends beyond homeostasis. When primed IEL isolated from infected mice are adoptively transferred into naïve mice, recipients experience reduced mortality rates after lethal parasite challenge (Lepage et al., 1998; Buzoni-Gatel et al., 1999). The protective effects are recapitulated when CD8*αβ* expressing IEL are adoptively transferred, although *γδ* ^+^ T cells also contribute to protection (Lepage et al., 1998). Primed CD8*αβ* ^+^ IEL were also described as possessing antigen specific cytotoxic activity and producing IFN-*γ*. Thus, it is likely that CD8^+^ IEL kill *T. gondii* infected epithelial cells and contribute to type I immunity. Further studies using CCR2^−/−^ mice showed that although they succumb to oral challenge with *T. gondii*, knockout animals exhibit significantly less intestinal pathology compared to WT mice (Egan et al., 2009). The decrease in immunopathology was attributed to lack of retention of CD103^+^ IEL, and adoptive transfer of wildtype IEL resulted in both improved survival and more severe intestinal pathology (Egan et al., 2009). Thus, although the IEL compartment contributes to protective *Toxoplasma* immunity, it can also play a role in initiating damage and inflammation in the small intestine.

### Innate Lymphoid Cells

Innate lymphoid cells (ILCs) are a newly described family of immune cells comprised of three subsets (ILC1, 2, and 3). They are prominent at mucosal interfaces including the small intestine (Spits and Cupedo, 2012; Bennett et al., 2015). ILC1 produce IFN-*γ*, ILC2 are associated with Th2-like cytokines, and ILC3 produce IL17 and IL-22 (Spits et al., 2013; Artis and Spits, 2015; Eberl et al., 2015). It remains unclear how each contribute to mucosal anti-*Toxoplasma* immunity. There is evidence that T-bet^+^ ILC1 secrete IFN-*γ* in response to oral inoculation with *T. gondii*; however, the contribution appears minor compared to CD4^+^ T cells. Tbx21^−/−^ mice, which lack ILC1, generate a strong IFN-*γ* response driven by T-bet independent CD4^+^ T cells (Lopez-Yglesias et al., 2018). Nevertheless, other studies found that ILC-like cells are protective against *T. gondii* infection (Klose et al., 2014). It was found that ROR*γ*t^+^ ILC3 frequencies decrease during *T. gondii* infection, and it was suggested that these cells play a role in limiting T cell responses and pathology during *T. gondii* infection (Wagage et al., 2015). Further studies are necessary to discern the role the ILC compartment plays during intestinal *T. gondii* infection.

### Dendritic Cells and Inflammatory Monocytes

Dendritic cells (DCs) are widely regarded as being pivotal in activation of T cell immunity, as well as playing a crucial role in maintenance of tolerance in the intestinal environment (Stagg, 2018; Sun et al., 2020). In the gut, discrete DC subsets can be identified based upon expression of CD11b and CD103 (Persson et al., 2013). Because of a requirement for transcription factor IRF8, the CD11b^-^CD103^+^ subset of lamina propria DC are likely related to splenic CD8*α* ^+^ DC that produce IL-12 and mediate protection in i. p. models of *T. gondii* infection (Edelson et al., 2010; Cohen et al., 2014). Essentially all of the IL-12 produced by CD11b^-^CD103^+^ lamina propria DC comes from non-infected cells (Cohen and Denkers, 2015). This suggests that these cells respond to parasite molecules present in the extracellular environment, or that the cells have been injected with parasite effector proteins as is known to occur for *Toxoplasma* rhoptry proteins (Koshy et al., 2012; Chen et al., 2020). Alternatively, it is possible DC responses are initiated by host-derived alarm signals triggered by infection.

Oral infection with *Toxoplasma* elicits a large influx of inflammatory monocytes into the lamina propria (Cohen and Denkers, 2015). Recruitment of these cells, whose presence is dependent upon chemokine receptor CCR2, mediates resistance to *Toxoplasma* as the parasite enters the intestinal mucosa (Dunay et al., 2008). Inflammatory monocytes in the lamina propria express IL-12, and it is possible that they play a role in promoting induction of protective Th1 cells (Cohen et al., 2013). Nevertheless, inflammatory monocytes are recruited into the intestine concomitant with appearance of Th1 effectors. Therefore, these cells may be more important as executioners of IFN-γ-dependent control of *T. gondii* using mechanisms such as iNOS/NOS2 and the IRG effector family that destroy the parasitophorous vacuole membrane (Khan et al., 1997; Hunn et al., 2011; Wang S. et al., 2019).

### Neutrophils

Neutrophils are among the first immune cells to infiltrate the site of infection. Within three days of oral inoculation with *T. gondii*, a rapid influx into the small intestine lamina propria is observed (Sukhumavasi et al., 2008; Gregg et al., 2013). Additionally, it has been reported that *T. gondii* preferentially infects infiltrating neutrophils to disseminate into other host tissues (Coombes et al., 2013). Activated neutrophils can secrete cytokines important for type I immunity including IL-12 and TNF-α, and they deploy neutrophil extracellular traps that ensnare and kill extracellular tachyzoites (Bliss et al., 1999; Bliss et al., 2000; Bliss et al., 2001; Sukhumavasi et al., 2008; Abi Abdallah et al., 2012). Whether these mechanisms operate within the intestinal mucosa during *T. gondii* infection is not known. The role neutrophils play in inducing intestinal immunity and pathology also remains unclear. In one study wildtype mice depleted of neutrophils with monoclonal antibody treatment survived the infection and displayed similar intestinal pathology compared to untreated mice. In the same study, CCR2^−/−^ mice depleted of neutrophils exhibited less intestinal tissue damage compared to untreated CCR2^−/−^ mice (Dunay et al., 2010). In an intraperitoneal infection model, depletion of neutrophils within the first four days of infection resulted in mortality associated with severe lesions and increased systemic parasite burdens, and a similar result was seen with CXCR2 knockout mice that are defective in neutrophil recruitment (Bliss et al., 2001; Del Rio et al., 2001). These disparate results might be explained by different routes of infection or different effectiveness of antibody depletion protocols. Further studies are required to discern the role neutrophils play in intestinal anti-*Toxoplasma* immunity.

### T Cells

*T. gondii* is a well-known inducer of type I immunity that during oral infection includes an expansion of parasite and microbiota-specific Th1 cells (Hand et al., 2012). Myeloid cell derived IL-12 is the primary driver of type I immunity (Gazzinelli et al., 1994; Yap et al., 2000). Although Th1 immunity generated in response to *Toxoplasma* is required to survive infection, it also underlies the severe intestinal immunopathology that can occur during infection (Liesenfeld et al., 1996; Raetz et al., 2013b; Burger et al., 2018). Interestingly, a robust IFN-*γ* ^+^ CD4^+^ T cell response was observed in Tbx21^−/−^ mice that lack expression of T-bet, regarded as the master regulator of Th1 differentiation. This clearly indicates that the anti-*Toxoplasma* Th1 response and Th1-mediated intestinal damage can be elicited without T-bet (Lopez-Yglesias et al., 2018).

Although the Th1 CD4^+^ T cell response dominates during *T. gondii* infection, Th17 cells have also been shown to play a role in *T. gondii* immunity. Mice lacking class I-restricted T cell-associated molecule (CRTAM) expression on T cells have fewer IL-17a and IL-22-secreting Th17 cells. This is associated with decreased antimicrobial peptide production, and increased pathology and microbial translocation into systemic tissues after oral inoculation with *T. gondii* (Cortez et al., 2014; Cervantes-Barragan et al., 2019). These data highlight emerging roles for Th17 T cells in controlling *T. gondii* induced intestinal dysbiosis, systemic dissemination of intestinal microbes, and immunopathology.

Regulatory T cells (T_reg_) are well-known to possess an important function in controlling proinflammatory tissue damage in the intestinal mucosa, in large part through production of IL-10 (Neumann et al., 2019). During *Toxoplasma* infection, the T_reg_ population rapidly disappears which likely plays a role in inflammatory pathology induced by the parasite (Oldenhove et al., 2009). In part, this is due to conversion of Treg cells into T-bet^+^ IFN-*γ* producing cells. Nevertheless, the collapse in the intestinal T_reg_ population appears to have a multifactorial root cause insofar as deprivation of IL-2 associated with massive Th1 expansion also underlies this phenomenon (Benson et al., 2012).

### B Cells

B cells are present in large number in both the lamina propria and Peyer’s patches of the small intestine (Gregg et al., 2013; Reboldi and Cyster, 2016). Following oral infection with type II strain cysts, µMT mice (which lack B cells) survive acute infection, but eventually succumb during the chronic stage 3–4 weeks later (Kang et al., 2000). In an oral inoculation model following i. p. vaccination with an attenuated *T. gondii* strain, µMT animals survive lethal challenge in a manner indistinguishable from wildtype vaccinated controls (Johnson et al., 2004). However, during i. p. infection with highly virulent type I parasites, vaccinated µMT mice succumb to lethal challenge (Sayles et al., 2000). Taken together, while B cells may have a role in i. p. vaccination-induced immunity, they appear less important in the context of protection in the intestinal mucosa.

## Conclusions and Future Directions

While our understanding of pathogenesis of *Toxoplasma* infection in the intestinal mucosa has expanded significantly in recent decades, there are still areas requiring exploration. The precise events in early innate immune triggering remain shrouded in mystery. The exact relationship between parasite-derived signals, host-derived signals and possibly host damage-associated danger signals remains to be clarified. Along similar lines, the precise sequence of events that lead to immunopathology in the intestine remains obscure. Also largely unexplored is how the mucosal immune system remembers and responds to secondary infection with *Toxoplasma*, an area that has significant relevance to issues of vaccine development not only to *T. gondii*, but to orally acquired microbial pathogens as a whole.

## Author Contributions

ED and LS conceived and wrote the manuscript. All authors contributed to the article and approved the submitted version.

## Conflict of Interest

The authors declare that the research was conducted in the absence of any commercial or financial relationships that could be construed as a potential conflict of interest.

## References

[B1] Abi Abdallah D. S. Lin C. Ball C. J. King M. R. Duhamel G. E. Denkers E. Y. (2012). Toxoplasma gondii triggers release of human and mouse neutrophil extracellular traps. Infect. Immun. 80 (2), 768–777. 10.1128/IAI.05730-11 22104111 PMC3264325

[B2] Allaire J. M. Crowley S. M. Law H. T. Chang S. Y. Ko H. J. Vallance B. A. (2018). The Intestinal Epithelium: Central Coordinator of Mucosal Immunity. Trends Immunol. 39 (9), 677–696. 10.1016/j.it.2018.04.002 29716793

[B3] Artis D. Spits H. (2015). The biology of innate lymphoid cells. Nature 517 (7534), 293–301. 10.1038/nature14189 25592534

[B4] Barragan A. Sibley L. D. (2002). Transepithelial migration of *Toxoplasma gondii* is linked to parasite migration and virulence. J. Exp. Med. 195, 1625–1633. 10.1084/jem.20020258 12070289 PMC2193562

[B5] Barragan A. Sibley L. D. (2003). Migration of *Toxoplasma gondii* across biological barriers. Trends Microbiol. 11 (9), 426–430. 10.1016/s0966-842x(03)00205-1 13678858

[B6] Barragan A. Brossier F. Sibley L. D. (2005). Transepithelial migration of *Toxoplasma gondii* involves an interaction of intercellular adhesion molecule 1 (ICAM-1) with parasite adhesin MIC2. Cell Microbiol. 7, 561–568. 10.1111/j.1462-5822.2005.00486.x 15760456

[B7] Bennett M. S. Round J. L. Leung D. T. (2015). Innate-like lymphocytes in intestinal infections. Curr. Opin. Infect. Dis. 28 (5), 457–463. 10.1097/QCO.0000000000000189 26270655 PMC4925623

[B8] Benson A. Pifer R. Behrendt C. L. Hooper L. V. Yarovinsky F. (2009). Gut commensal bacteria direct a protective immune response against Toxoplasma gondii. Cell Host Microbe 6 (2), 187–196. 10.1016/j.chom.2009.06.005 19683684 PMC2746820

[B9] Benson A. Murray S. Divakar P. Burnaevskiy N. Pifer R. Forman J. (2012). Microbial Infection-Induced Expansion of Effector T Cells Overcomes the Suppressive Effects of Regulatory T Cells via an IL-2 Deprivation Mechanism. J. Immunol. 188, 800–810. 10.4049/jimmunol.1100769 22147768 PMC3253229

[B10] Bliss S. K. Marshall A. J. Zhang Y. Denkers E. Y. (1999). Human polymorphonuclear leukocytes produce IL-12, TNF-a, and the chemokines macrophage-inflammatory protein-1a and -1b in response to *Toxoplasma gondii* antigens. J. Immunol. 162, 7369–7375. 10358188

[B11] Bliss S. K. Butcher B. A. Denkers E. Y. (2000). Rapid recruitment of neutrophils with prestored IL-12 during microbial infection. J. Immunol. 165, 4515–4521. 10.4049/jimmunol.165.8.4515 11035091

[B12] Bliss S. K. Gavrilescu L. C. Alcaraz A. Denkers E. Y. (2001). Neutrophil depletion during Toxoplasma gondii infection leads to impaired immunity and lethal systemic pathology. Infect. Immun. 69 (8), 4898–4905. 10.1128/IAI.69.8.4898-4905.2001 11447166 PMC98580

[B13] Braun L. Brenier-Pinchart M. P. Yogavel M. Curt-Varesano A. Curt-Bertini R. L. Hussain T. (2013). A Toxoplasma dense granule protein, GRA24, modulates the early immune response to infection by promoting a direct and sustained host p38 MAPK activation. J. Exp. Med. 210 (10), 2071–2086. 10.1084/jem.20130103 24043761 PMC3782045

[B14] Briceno M. P. Nascimento L. A. Nogueira N. P. Barenco P. V. Ferro E. A. Rezende-Oliveira K. (2016). Toxoplasma gondii Infection Promotes Epithelial Barrier Dysfunction of Caco-2 Cells. J. Histochem. Cytochem. 64 (8), 459–469. 10.1369/0022155416656349 27370796 PMC4971781

[B15] Burger E. Araujo A. Lopez-Yglesias A. Rajala M. W. Geng L. Levine B. (2018). Loss of Paneth Cell Autophagy Causes Acute Susceptibility to Toxoplasma gondii-Mediated Inflammation. Cell Host Microbe 23 (2), 177–90.e4. 10.1016/j.chom.2018.01.001 29358083 PMC6179445

[B16] Buzoni-Gatel D. Debbabi H. Moretto M. Dimier-Poisson I. H. Lepage A. C. Bout D. T. (1999). Intraepithelial lymphocytes traffic to the intestine and enhance resistance to Toxoplasma gondii oral infection. J. Immunol. 162 (10), 5846–5852. 10229819

[B17] Cervantes-Barragan L. Cortez V. S. Wang Q. McDonald K. G. Chai J. N. Di Luccia B. (2019). CRTAM Protects Against Intestinal Dysbiosis During Pathogenic Parasitic Infection by Enabling Th17 Maturation. Front. Immunol. 10, 1423. 10.3389/fimmu.2019.01423 31312200 PMC6614434

[B18] Chen L. Christian D. A. Kochanowsky J. A. Phan A. T. Clark J. T. Wang S. (2020). The Toxoplasma gondii virulence factor ROP16 acts in cis and trans, and suppresses T cell responses. J. Exp. Med. 217 (3), e20181757. 10.1084/jem.20181757 PMC7062521 31961916

[B19] Cheroutre H. Lambolez F. Mucida D. (2011). The light and dark sides of intestinal intraepithelial lymphocytes. Nat. Rev. Immunol. 11 (7), 445–456. 10.1038/nri3007 21681197 PMC3140792

[B20] Cohen S. B. Denkers E. Y. (2015). Impact of Toxoplasma gondii on Dendritic Cell Subset Function in the Intestinal Mucosa. J. Immunol. 195 (6), 2754–2762. 10.4049/jimmunol.1501137 26283477 PMC4561193

[B21] Cohen S. B. Maurer K. J. Egan C. E. Oghumu S. Satoskar A. R. Denkers E. Y. (2013). CXCR3-dependent CD4(+) T cells are required to activate inflammatory monocytes for defense against intestinal infection. PLoS Pathog. 9 (10), e1003706. 10.1371/journal.ppat.1003706 24130498 PMC3795032

[B22] Cohen S. B. Smith N. L. McDougal C. Pepper M. Shah S. Yap G. S. (2014). beta-Catenin Signaling Drives Differentiation and Proinflammatory Function of IRF8-Dependent Dendritic Cells. J. Immunol. 194, 210–222. 10.4049/jimmunol.1402453 PMC4333072 25416805

[B23] Coombes J. L. Charsar B. A. Han S. J. Halkias J. Chan S. W. Koshy A. A. (2013). Motile invaded neutrophils in the small intestine of Toxoplasma gondii-infected mice reveal a potential mechanism for parasite spread. Proc. Natl. Acad. Sci. U. S. A. 110 (21), E1913–E1922. 10.1073/pnas.1220272110 23650399 PMC3666704

[B24] Cortez V. S. Cervantes-Barragan L. Song C. Gilfillan S. McDonald K. G. Tussiwand R. (2014). CRTAM controls residency of gut CD4+CD8+ T cells in the steady state and maintenance of gut CD4+ Th17 during parasitic infection. J. Exp. Med. 211 (4), 623–633. 10.1084/jem.20130904 24687959 PMC3978276

[B25] Courret N. Darche S. Sonigo P. Milon G. Buzoni-Gatel D. Tardieux I. (2006). CD11c and CD11b expressing mouse leukocytes transport single *Toxoplasma gondii* tachyzoites to the brain. Blood 107, 309–316. 10.1182/blood-2005-02-0666 16051744 PMC1895351

[B26] Craven M. Egan C. E. Dowd S. E. McDonough S. P. Dogan B. Denkers E. Y. (2012). Inflammation drives dysbiosis and bacterial invasion in murine models of ileal Crohn’s disease. PLoS One 7 (7), e41594. 10.1371/journal.pone.0041594 22848538 PMC3404971

[B27] de Carvalho R. V. H. Zamboni D. S. (2020). Inflammasome Activation in Response to Intracellular Protozoan Parasites. Trends Parasitol. 36 (5), 459–472. 10.1016/j.pt.2020.02.006 32298633

[B28] Del Rio L. Bennouna S. Salinas J. Denkers E. Y. (2001). CXCR2 deficiency confers impaired neutrophil recruitment and increased susceptibility during Toxoplasma gondii infection. J. Immunol. 167 (11), 6503–6509. 10.4049/jimmunol.167.11.6503 11714818

[B29] Denkers E. Y. Gazzinelli R. T. (1998). Regulation and function of T-cell-mediated immunity during Toxoplasma gondii infection. Clin. Microbiol. Rev. 11 (4), 569–588. 10.1128/CMR.11.4.569 9767056 PMC88897

[B30] Denkers E. Y. Bzik D. J. Fox B. A. Butcher B. A. (2012). An inside job: hacking into Janus kinase/signal transducer and activator of transcription signaling cascades by the intracellular protozoan Toxoplasma gondii. Infect. Immun. 80 (2), 476–482. 10.1128/IAI.05974-11 22104110 PMC3264314

[B31] Dubey J. P. (2013). “The history and life-cycle of Toxoplasma gondii,” in Toxoplasma gondii the model apicomplexan: perspective and methods, 2nd ed. Eds. Weiss L. M. Kim K. (San Diego: Academic Press), 1–17.

[B32] Dunay I. R. Damatta R. A. Fux B. Presti R. Greco S. Colonna M. (2008). Gr1(+) Inflammatory Monocytes Are Required for Mucosal Resistance to the Pathogen Toxoplasma gondii. Immunity 29 (2), 306–317. 10.1016/j.immuni.2008.05.019 18691912 PMC2605393

[B33] Dunay I. R. Fuchs A. Sibley L. D. (2010). Inflammatory monocytes but not neutrophils are necessary to control infection with Toxoplasma gondii in mice. Infect. Immun. 78 (4), 1564–1570. 10.1128/IAI.00472-09 20145099 PMC2849397

[B34] Dupont C. D. Christian D. A. Hunter C. A. (2012). Immune response and immunopathology during toxoplasmosis. Semin. Immunopathol. 34 (6), 793–813. 10.1007/s00281-012-0339-3 22955326 PMC3498595

[B35] Eberl G. Colonna M. Di Santo J. P. McKenzie A. N. (2015). Innate lymphoid cells. Innate lymphoid cells: a new paradigm in immunology. Science 348 (6237), aaa6566. 10.1126/science.aaa6566 25999512 PMC5658207

[B36] Edelson B. T. Kc W. Juang R. Kohyama M. Benoit L. A. Klekotka P. A. (2010). Peripheral CD103+ dendritic cells form a unified subset developmentally related to CD8alpha+ conventional dendritic cells. J. Exp. Med. 207 (4), 823–836. 10.1084/jem.20091627 20351058 PMC2856032

[B37] Egan C. E. Craven M. D. Leng J. Mack M. Simpson K. W. Denkers E. Y. (2009). CCR2-dependent intraepithelial lymphocytes mediate inflammatory gut pathology during Toxoplasma gondii infection. Mucosal Immunol. 2 (6), 527–535. 10.1038/mi.2009.105 19741601 PMC2860785

[B38] Egan C. E. Cohen S. B. Denkers E. Y. (2011a). Insights into inflammatory bowel disease using Toxoplasma gondii as an infectious trigger. Immunol. Cell Biol. 90, 668–675. 10.1038/icb.2011.93 22064707 PMC4094106

[B39] Egan C. E. Maurer K. J. Cohen S. B. Mack M. Simpson K. W. Denkers E. Y. (2011b). Synergy between intraepithelial lymphocytes and lamina propria T cells drives intestinal inflammation during infection. Mucosal Immunol. 4, 658–670. 10.1038/mi.2011.31 21796113 PMC3196813

[B40] Ewald S. E. Chavarria-Smith J. Boothroyd J. C. (2014). NLRP1 is an inflammasome sensor for Toxoplasma gondii. Infect. Immun. 82 (1), 460–468. 10.1128/IAI.01170-13 24218483 PMC3911858

[B41] Gazzinelli R. T. Denkers E. Y. (2006). Protozoan encounters with Toll-like receptor signalling pathways: implications for host parasitism. Nat. Rev. Immunol. 6 (12), 895–906. 10.1038/nri1978 17110955

[B42] Gazzinelli R. T. Wysocka M. Hayashi S. Denkers E. Y. Hieny S. Caspar P. (1994). Parasite-induced IL-12 stimulates early IFN-gamma synthesis and resistance during acute infection with Toxoplasma gondii. J. Immunol. 153 (6), 2533–2543. 7915739

[B43] Gazzinelli R. T. Wysocka M. Hieny S. Scharton-Kersten T. Cheever A. Kuhn R. (1996). In the absence of endogenous IL-10, mice acutely infected with Toxoplasma gondii succumb to a lethal immune response dependent on CD4+ T cells and accompanied by overproduction of IL-12, IFN-gamma and TNF-alpha. J. Immunol. 157 (2), 798–805. 8752931

[B44] Gazzinelli R. T. Mendonca-Neto R. Lilue J. Howard J. Sher A. (2014). Innate resistance against Toxoplasma gondii: an evolutionary tale of mice, cats, and men. Cell Host Microbe 15 (2), 132–138. 10.1016/j.chom.2014.01.004 24528860 PMC4006104

[B45] Gorfu G. Cirelli K. M. Melo M. B. Mayer-Barber K. Crown D. Koller B. H. (2014). Dual role for inflammasome sensors NLRP1 and NLRP3 in murine resistance to Toxoplasma gondii. MBio 5 (1), e01117–13. 10.1128/mBio.01117-13 PMC3944820 24549849

[B46] Gov L. Karimzadeh A. Ueno N. Lodoen M. B. (2013). Human innate immunity to Toxoplasma gondii is mediated by host caspase-1 and ASC and parasite GRA15. MBio 4 (4), e00255–13. 10.1128/mBio.00255-13 PMC3705447 23839215

[B47] Gov L. Schneider C. A. Lima T. S. Pandori W. Lodoen M. B. (2017). NLRP3 and Potassium Efflux Drive Rapid IL-1beta Release from Primary Human Monocytes during Toxoplasma gondii Infection. J. Immunol. 199 (8), 2855–2864. 10.4049/jimmunol.1700245 28904126 PMC5648586

[B48] Gregg B. Taylor B. C. John B. Tait-Wojno E. D. Girgis N. M. Miller N. (2013). Replication and distribution of Toxoplasma gondii in the small intestine after oral infection with tissue cysts. Infect. Immun. 81 (5), 1635–1643. 10.1128/IAI.01126-12 23460516 PMC3647985

[B49] Guy-Grand D. Cerf-Bensussan N. Malissen B. Malassis-Seris M. Briottet C. Vassalli P. (1991). Two gut intraepithelial CD8+ lymphocyte populations with different T cell receptors: a role for the gut epithelium in T cell differentiation. J. Exp. Med. 173 (2), 471–481. 10.1084/jem.173.2.471 1824857 PMC2118788

[B50] Hand T. W. Dos Santos L. M. Bouladoux N. Molloy M. J. Pagan A. J. Pepper M. (2012). Acute Gastrointestinal Infection Induces Long-Lived Microbiota-Specific T Cell Responses. Science 337, 1553–1556. 10.1126/science.1220961 22923434 PMC3784339

[B51] Heimesaat M. M. Bereswill S. Fischer A. Fuchs D. Struck D. Niebergall J. (2006). Gram-Negative Bacteria Aggravate Murine Small Intestinal Th1-Type Immunopathology following Oral Infection with Toxoplasma gondii. J. Immunol. 177 (12), 8785–8795. 10.4049/jimmunol.177.12.8785 17142781

[B52] Heimesaat M. M. Fischer A. Jahn H. K. Niebergall J. Freudenberg M. Blaut M. (2007). Exacerbation of Murine Ileitis By Toll-Like Receptor 4 Meditated Sensing of Lipopolysaccharide From Commensal Escherichia coli. Gut 56, 941–948. 10.1136/gut.2006.104497 17255219 PMC1994376

[B53] Hunn J. P. Feng C. G. Sher A. Howard J. C. (2011). The immunity-related GTPases in mammals: a fast-evolving cell-autonomous resistance system against intracellular pathogens. Mamm. Genome 22 (1-2), 43–54. 10.1007/s00335-010-9293-3 21052678 PMC3438224

[B54] Hunter C. A. Sibley L. D. (2012). Modulation of innate immunity by Toxoplasma gondii virulence effectors. Nat. Rev. Microbiol. 10 (11), 766–778. 10.1038/nrmicro2858 23070557 PMC3689224

[B55] Jensen K. D. Wang Y. Wojno E. D. Shastri A. J. Hu K. Cornel L. (2011). Toxoplasma polymorphic effectors determine macrophage polarization and intestinal inflammation. Cell Host Microbe 9 (6), 472–483. 10.1016/j.chom.2011.04.015 21669396 PMC3131154

[B56] Jensen K. D. Hu K. Whitmarsh R. J. Hassan M. A. Julien L. Lu D. (2013). Toxoplasma rhoptry kinase ROP16 promotes host resistance to oral infection and intestinal inflammation only in the context of the dense granule protein GRA15. Infect. Immun. 81, 2156–2167. 10.1128/iai.01185-12 23545295 PMC3676013

[B57] Johnson L. L. Lanthier P. Hoffman J. Chen W. (2004). Vaccination protects B cell-deficient mice against an oral challenge with mildly virulent Toxoplasma gondii. Vaccine 22 (29-30), 4054–4061. 10.1016/j.vaccine.2004.03.056 15364456

[B58] Kang H. Remington J. S. Suzuki Y. (2000). Decreased resistance of B cell-deficient mice to infection with Toxoplasma gondii despite unimpaired expression of IFN-gamma, TNF-alpha, and inducible nitric oxide synthase. J. Immunol. 164 (5), 2629–2634. 10.4049/jimmunol.164.5.2629 10679102

[B59] Kayama H. Takeda K. (2012). Regulation of intestinal homeostasis by innate and adaptive immunity. Int. Immunol. 24 (11), 673–680. 10.1093/intimm/dxs094 22962437

[B60] Khan I. A. Schwartzman J. D. Matsuura T. Kasper L. H. (1997). A dichotomous role for nitric oxide during acute *Toxoplasma gondii* infection in mice. Proc. Natl. Acad. Sci. U. S. A. 94, 13955–13960. 10.1073/pnas.94.25.13955 9391134 PMC28414

[B61] Kim L. Del Rio L. Butcher B. A. Mogensen T. H. Paludan S. Flavell R. A. (2005). p38 MAPK autophosphorylation drives macrophage IL-12 production during intracellular infection. J. Immunol. 174, 4178–4184. 10.4049/jimmunol.174.7.4178 15778378

[B62] Klose C. S. Flach M. Mohle L. Rogell L. Hoyler T. Ebert K. (2014). Differentiation of type 1 ILCs from a common progenitor to all helper-like innate lymphoid cell lineages. Cell 157 (2), 340–356. 10.1016/j.cell.2014.03.030 24725403

[B63] Koblansky A. A. Jankovic D. Oh H. Hieny S. Sungnak W. Mathur R. (2012). Recognition of Profilin by Toll-like Receptor 12 Is Critical for Host Resistance to Toxoplasma gondii. Immunity 38, 119–130. 10.1016/j.immuni.2012.09.016 23246311 PMC3601573

[B64] Koshy A. A. Dietrich H. K. Christian D. A. Melehani J. H. Shastri A. J. Hunter C. A. (2012). Toxoplasma Co-opts Host Cells It Does Not Invade. PLoS Pathog. 8 (7), e1002825. 10.1371/journal.ppat.1002825 22910631 PMC3406079

[B65] Kucera K. Koblansky A. A. Saunders L. P. Frederick K. B. De La Cruz E. M. Ghosh S. (2010). Structure-based analysis of Toxoplasma gondii profilin: a parasite-specific motif is required for recognition by Toll-like receptor 11. J. Mol. Biol. 403 (4), 616–629. 10.1016/j.jmb.2010.09.022 20851125 PMC2957522

[B66] Lepage A. C. Buzoni-Gatel D. Bout D. T. Kasper L. H. (1998). Gut-derived intraepithelial lymphocytes induce long term immunity against Toxoplasma gondii. J. Immunol. 161 (9), 4902–4908. 9794424

[B67] Liesenfeld O. Kosek J. Remington J. S. Suzuki Y. (1996). Association of CD4^+^ T cell-dependent, IFN-g-mediated necrosis of the small intestine with genetic susceptibility of mice to peroral infection with *Toxoplasma gondii* . J. Exp. Med. 184, 597–607. 10.1084/jem.184.2.597 8760813 PMC2192709

[B68] Liesenfeld O. Kang H. Park D. Nguyen T. A. Parkhe C. V. Watanabe H. (1999). TNF-a, nitric oxide and IFN-g are all critical for development of necrosis in the small intestine and early mortality in genetically susceptible mice infected perorally with *Toxoplasma gondii* . Parasite Immunol. 21, 365–376. 10.1046/j.1365-3024.1999.00237.x 10417671

[B69] Lopez-Yglesias A. H. Burger E. Araujo A. Martin A. T. Yarovinsky F. (2018). T-bet-independent Th1 response induces intestinal immunopathology during Toxoplasma gondii infection. Mucosal Immunol. 11 (3), 921–931. 10.1038/mi.2017.102 29297501 PMC6179443

[B70] Lopez-Yglesias A. H. Camanzo E. Martin A. T. Araujo A. M. Yarovinsky F. (2019). TLR11-independent inflammasome activation is critical for CD4+ T cell-derived IFN-gamma production and host resistance to Toxoplasma gondii. PLoS Pathog. 15 (6), e1007872. 10.1371/journal.ppat.1007872 31194844 PMC6599108

[B71] Luu L. Coombes J. L. (2015). Dynamic two-photon imaging of the immune response to Toxoplasma gondii infection. Parasite Immunol. 37 (3), 118–126. 10.1111/pim.12161 25407960

[B72] Martorelli Di Genova B. Wilson S. K. Dubey J. P. Knoll L. J. (2019). Intestinal delta-6-desaturase activity determines host range for Toxoplasma sexual reproduction. PLoS Biol. 17 (8), e3000364. 10.1371/journal.pbio.3000364 31430281 PMC6701743

[B73] McCabe R. Remington J. S. (1988). Toxoplasmosis: The time has come. N. Eng. J. Med. 380, 313–315. 10.1056/NEJM198802043180509 3336423

[B74] McLeod R. M. van Tubbergen C. Montoya J. G. Petersen E. (2013). “Human Toxoplasma infection,” in Toxoplasma gondii The Model Apicomplexan: Perspectives and Methods, 2nd ed. Eds. Weiss L. M. Kim K. (San Diego: Academic Press), 100–159.

[B75] Mercer H. L. Snyder L. M. Doherty C. M. Fox B. A. Bzik D. J. Denkers E. Y. (2020). Toxoplasma gondii dense granule protein GRA24 drives MyD88-independent p38 MAPK activation, IL-12 production and induction of protective immunity. PloS Pathog. 16 (5), e1008572. 10.1371/journal.ppat.1008572 32413093 PMC7255617

[B76] Minns L. A. Menard L. C. Foureau D. M. Darche S. Ronet C. Mielcarz D. W. (2006). TLR9 Is Required for the Gut-Associated Lymphoid Tissue Response following Oral Infection of Toxoplasma gondii. J. Immunol. 176 (12), 7589–7597. 10.4049/jimmunol.176.12.7589 16751405

[B77] Molloy M. J. Grainger J. R. Bouladoux N. Hand T. W. Koo L. Y. Naik S. (2013). Intraluminal containment of commensal outgrowth in the gut during infection-induced dysbiosis. Cell Host Microbe 14 (3), 318–328. 10.1016/j.chom.2013.08.003 24034617 PMC4806337

[B78] Mukhopadhyay D. Arranz-Solis D. Saeij J. P. J. (2020). Toxoplasma GRA15 and GRA24 are important activators of the host innate immune response in the absence of TLR11. PLoS Pathog. 16 (5), e1008586. 10.1371/journal.ppat.1008586 32453782 PMC7274473

[B79] Neumann C. Scheffold A. Rutz S. (2019). Functions and regulation of T cell-derived interleukin-10. Semin. Immunol. 44, 101344. 10.1016/j.smim.2019.101344 31727465

[B80] Nutsch K. M. Hsieh C. S. (2012). T cell tolerance and immunity to commensal bacteria. Curr. Opin. Immunol. 24 (4), 385–391. 10.1016/j.coi.2012.04.009 22613090 PMC3423487

[B81] Oldenhove G. Bouladoux N. Wohlfert E. A. Hall J. A. Chou D. Dos Santos L. (2009). Decrease of Foxp3(+) Treg Cell Number and Acquisition of Effector Cell Phenotype during Lethal Infection. Immunity 31, 772–786. 10.1016/j.immuni.2009.10.001 19896394 PMC2814877

[B82] Persson E. K. Scott C. L. Mowat A. M. Agace W. W. (2013). Dendritic cell subsets in the intestinal lamina propria: ontogeny and function. Eur. J. Immunol. 43 (12), 3098–3107. 10.1002/eji.201343740 23966272 PMC3933733

[B83] Pfaff A. W. Liesenfeld O. Candolfi E. (2007). “Congenital toxoplasmosis,” in Toxoplasma molecular and cellular biology. Eds. Ajioka J. W. Soldati D. (Norfolk: Horizon Bioscience), 93–110.

[B84] Quan J. H. Huang R. Wang Z. Huang S. Choi I. W. Zhou Y. (2018). P2X7 receptor mediates NLRP3-dependent IL-1beta secretion and parasite proliferation in Toxoplasma gondii-infected human small intestinal epithelial cells. Parasit. Vectors 11 (1), 1. 10.1186/s13071-017-2573-y 29291748 PMC5748956

[B85] Raetz M. Kibardin A. Sturge C. R. Pifer R. Li H. Burstein E. (2013a). Cooperation of TLR12 and TLR11 in the IRF8-dependent IL-12 response to Toxoplasma gondii profilin. J. Immunol. 191 (9), 4818–4827. 10.4049/jimmunol.1301301 24078692 PMC3805684

[B86] Raetz M. Hwang S. H. Wilhelm C. L. Kirkland D. Benson A. Sturge C. R. (2013b). Parasite-induced TH1 cells and intestinal dysbiosis cooperate in IFN-gamma-dependent elimination of Paneth cells. Nat. Immunol. 14 (2), 136–142. 10.1038/ni.2508 23263554 PMC3552073

[B87] Ramirez-Flores C. J. Cruz-Miron R. Lagunas-Cortes N. Mondragon-Castelan M. Mondragon-Gonzalez R. Gonzalez-Pozos S. (2020). Toxoplasma gondii excreted/secreted proteases disrupt intercellular junction proteins in epithelial cell monolayers to facilitate tachyzoites paracellular migration. Cell Microbiol. 1–9. 10.1111/cmi.13283 33108050

[B88] Reboldi A. Cyster J. G. (2016). Peyer’s patches: organizing B-cell responses at the intestinal frontier. Immunol. Rev. 271 (1), 230–245. 10.1111/imr.12400 27088918 PMC4835804

[B89] Rosowski E. E. Lu D. Julien L. Rodda L. Gaiser R. A. Jensen K. D. (2011). Strain-specific activation of the NF-kappaB pathway by GRA15, a novel Toxoplasma gondii dense granule protein. J. Exp. Med. 208 (1), 195–212. 10.1084/jem.20100717 21199955 PMC3023140

[B90] Safronova A. Araujo A. Camanzo E. T. Moon T. J. Elliott M. R. Beiting D. P. (2019). Alarmin S100A11 initiates a chemokine response to the human pathogen Toxoplasma gondii. Nat. Immunol. 20 (1), 64–72. 10.1038/s41590-018-0250-8 30455460 PMC6291348

[B91] Sansonetti P. J. (2011). To be or not to be a pathogen: that is the mucosally relevant question. Mucosal Immunol. 4 (1), 8–14. 10.1038/mi.2010.77 21150896

[B92] Sayles P. C. Gibson G. W. Johnson L. L. (2000). B cells are essential for vaccination-induced resistance to virulent Toxoplasma gondii. Infect. Immun. 68 (3), 1026–1033. 10.1128/iai.68.3.1026-1033.2000 10678903 PMC97244

[B93] Scanga C. A. Aliberti J. Jankovic D. Tilloy F. Bennouna S. Denkers E. Y. (2002). Cutting edge: MyD88 is required for resistance to *Toxoplasma gondii* infection and regulates parasite-induced IL-12 production by dendritic cells. J. Immunol. 168, 5997–6001. 10.4049/jimmunol.168.12.5997 12055206

[B94] Sher A. Coffman R. L. (1992). Regulation of immunity to parasites by T cells and T cell-dependent cytokines. Ann. Rev. Immunol. 10, 385–410. 10.1146/annurev.iy.10.040192.002125 1590992

[B95] Speer C. A. Dubey J. P. (1998). Ultrastructure of early stages of infections in mice fed Toxoplasma gondii oocysts. Parasitology 116 (Pt 1), 35–42. 10.1017/s0031182097001959 9481772

[B96] Spits H. Cupedo T. (2012). Innate lymphoid cells: emerging insights in development, lineage relationships, and function. Annu. Rev. Immunol. 30, 647–675. 10.1146/annurev-immunol-020711-075053 22224763

[B97] Spits H. Artis D. Colonna M. Diefenbach A. Di Santo J. P. Eberl G. (2013). Innate lymphoid cells–a proposal for uniform nomenclature. Nat. Rev. Immunol. 13 (2), 145–149. 10.1038/nri3365 23348417

[B98] Stagg A. J. (2018). Intestinal Dendritic Cells in Health and Gut Inflammation. Front. Immunol. 9, 2883. 10.3389/fimmu.2018.02883 30574151 PMC6291504

[B99] Sukhumavasi W. Egan C. E. Warren A. L. Taylor G. A. Fox B. A. Bzik D. J. (2008). TLR adaptor MyD88 is essential for pathogen control during oral toxoplasma gondii infection but not adaptive immunity induced by a vaccine strain of the parasite. J. Immunol. 181 (5), 3464–3473. 10.4049/jimmunol.181.5.3464 18714019 PMC2614926

[B100] Sun T. Nguyen A. Gommerman J. L. (2020). Dendritic Cell Subsets in Intestinal Immunity and Inflammation. J. Immunol. 204 (5), 1075–1083. 10.4049/jimmunol.1900710 32071090

[B101] van der Flier L. G. Clevers H. (2009). Stem cells, self-renewal, and differentiation in the intestinal epithelium. Annu. Rev. Physiol. 71, 241–260. 10.1146/annurev.physiol.010908.163145 18808327

[B102] Vester-Andersen M. K. Mirsepasi-Lauridsen H. C. Prosberg M. V. Mortensen C. O. Trager C. Skovsen K. (2019). Increased abundance of proteobacteria in aggressive Crohn’s disease seven years after diagnosis. Sci. Rep. 9 (1), 13473. 10.1038/s41598-019-49833-3 31530835 PMC6748953

[B103] Villeret B. Brault L. Couturier-Maillard A. Robinet P. Vasseur V. Secher T. (2013). Blockade of IL-1R signaling diminishes Paneth cell depletion and Toxoplasma gondii induced ileitis in mice. Am. J. Clin. Exp. Immunol. 2 (1), 107–116. 23885328 PMC3714202

[B104] Wagage S. Harms Pritchard G. Dawson L. Buza E. L. Sonnenberg G. F. Hunter C. A. (2015). The Group 3 Innate Lymphoid Cell Defect in Aryl Hydrocarbon Receptor Deficient Mice Is Associated with T Cell Hyperactivation during Intestinal Infection. PLoS One 10 (5), e0128335. 10.1371/journal.pone.0128335 26010337 PMC4444139

[B105] Wang S. El-Fahmawi A. Christian D. A. Fang Q. Radaelli E. Chen L. (2019). Infection-Induced Intestinal Dysbiosis Is Mediated by Macrophage Activation and Nitrate Production. MBio 10 (3), e00935–19. 10.1128/mBio.00935-19 PMC6538788 31138751

[B106] Wang Y. Cirelli K. M. Barros P. D. C. Sangare L. O. Butty V. Hassan M. A. (2019). Three Toxoplasma gondii Dense Granule Proteins Are Required for Induction of Lewis Rat Macrophage Pyroptosis. mBio 10 (1), e02388–18. 10.1128/mBio.02388-18 PMC6325250 30622189

[B107] Weight C. M. Carding S. R. (2012). The protozoan pathogen Toxoplasma gondii targets the paracellular pathway to invade the intestinal epithelium. Ann. N. Y. Acad. Sci. 1258, 135–142. 10.1111/j.1749-6632.2012.06534.x 22731726

[B108] Weiss L. M. Kim K. (2014). Toxoplasma gondii, the Model Apicomplexan: Perspectives and Methods. 2nd ed (Amsterdam: Academic Press).

[B109] Witola W. H. Mui E. Hargrave A. Liu S. Hypolite M. Montpetit A. (2011). NALP1 influences susceptibility to human congenital toxoplasmosis, proinflammatory cytokine response, and fate of Toxoplasma gondii-infected monocytic cells. Infect. Immun. 79 (2), 756–766. 10.1128/IAI.00898-10 21098108 PMC3028851

[B110] Yap G. Pesin M. Sher A. (2000). Cutting edge: IL-12 is required for the maintenance of IFN-gamma production in T cells mediating chronic resistance to the intracellular pathogen, Toxoplasma gondii. J. Immunol. 165 (2), 628–631. 10.4049/jimmunol.165.2.628 10878333

[B111] Yarovinsky F. Sher A. (2006). Toll-like receptor recognition of Toxoplasma gondii. Int. J. Parasitol. 36 (3), 255–259. 10.1016/j.ijpara.2005.12.003 16476433

[B112] Yarovinsky F. Zhang D. Anderson J. F. Bannenberg G. L. Serhan C. N. Hayden M. S. (2005). TLR11 activation of dendritic cells by a protozoan profilin-like protein. Science 308, 1626–1629. 10.1126/science.1109893 15860593

[B113] Zamboni D. S. Lima-Junior D. S. (2015). Inflammasomes in host response to protozoan parasites. Immunol. Rev. 265 (1), 156–171. 10.1111/imr.12291 25879291

[B114] Zhu F. Li C. Chu F. Tian X. Zhu J. (2020). Target Dysbiosis of Gut Microbes as a Future Therapeutic Manipulation in Alzheimer’s Disease. Front. Aging Neurosci. 12, 544235. 10.3389/fnagi.2020.544235 33132894 PMC7572848

